# Specific pattern of bone scan as a result of unintentional-intraarterial injection into the ulnar artery

**DOI:** 10.22088/cjim.13.2.439

**Published:** 2022

**Authors:** Amir Gholami, Iraj Jafaripour

**Affiliations:** 1 Department of Nuclear Medicine, Shahid Beheshti Hospital, Babol University of Medical Sciences, Babol, Iran; 2 Department of Cardiology, Ayatollah Rouhani Hospital, Babol University of Medical Sciences, Babol, Iran

**Keywords:** Bone scintigraphy, 99mTc-MDP, Intraarterial injection

## Abstract

**Background:**

Bone scintigraphy with 99mTc labeled radiopharmaceuticals is a valuable method in nuclear medicine for assessing the bony structure. In clinical setting, bone scintigraphy is accomplished after the injection of 99mTc labeled diphosphonate complexes into a peripheral vein. Inadvertent intra-arterial injection on the antecubital region may cause a special form of artifacts leading to problems inaccurately interpreting these studies as functional images.

**Case Presentation:**

We present a 44-year-old man with history of chest wall pain for bone scintigraphy as part of a work-up for determining the pain source. The patient received an injection of 740MBq 99mTc-methylene diphosphonate (MDP) into a blood vessel at the right forearm. Two hours later, an increased uptake of activity was observed on the right forearm and ulnar half of the wrist-hand in the whole body and spot images. The scan findings were consistent with the anatomical and physiological expectations of the ulnar arterial perfusion range. This case displays that an incidental injection with a 99mTc labeled diphosphonate into the ulnar artery results in a hot ulnar half of the palm and ulnar-sided three digits, because these areas are directly exposed to radiopharmaceutical, therefore more radiopharmaceutical is deposited.

**Conclusion:**

It is important for the physician, and especially for the nuclear medicine technician, to know the specific appearance created in the bone scan due to such an event so that they do not make a diagnostic mistake.

Bone scintigraphy with 99mTc labeled radiopharmaceuticals is a valuable method in nuclear medicine for assessing the bony structure. In clinical setting, bone scintigraphy is accomplished following the injection of 99mTc labeled diphosphonate complexes into a peripheral vein. Today bone scintigraphy is commonly used to assess a wide range of osseous abnormality from benign to malignant diseases. Bone scintigraphy can be performed as a multiphase study to evaluate regional blood flow, soft tissue hyperemia and finally delayed images for osseous abnormalities (1, 2). The blood flow images are dynamic sequences of planar images of the area of interest acquired as the radiopharmaceutical is administered. The early (blood pool or soft tissue period) images contain static planar images of the areas of interest, achieved instantly after the flow study and finished within 10 min following injection of the 99mTc labeled radiopharmaceuticals. Delayed images usually include the total body and are generally acquired 2–4 hours after injection. Inadvertent intra-arterial injection on the antecubital region may cause a special form of artifacts leading to problems inaccurately interpreting these studies as functional images. In these situations, there is an intense uptake in the extremity distal to the injection site on the images, in a specific pattern formerly defined as a hot forearm, a hot hand, or a glove phenomenon (3, 4, 5).

## Case Presentation

**Figure1:**
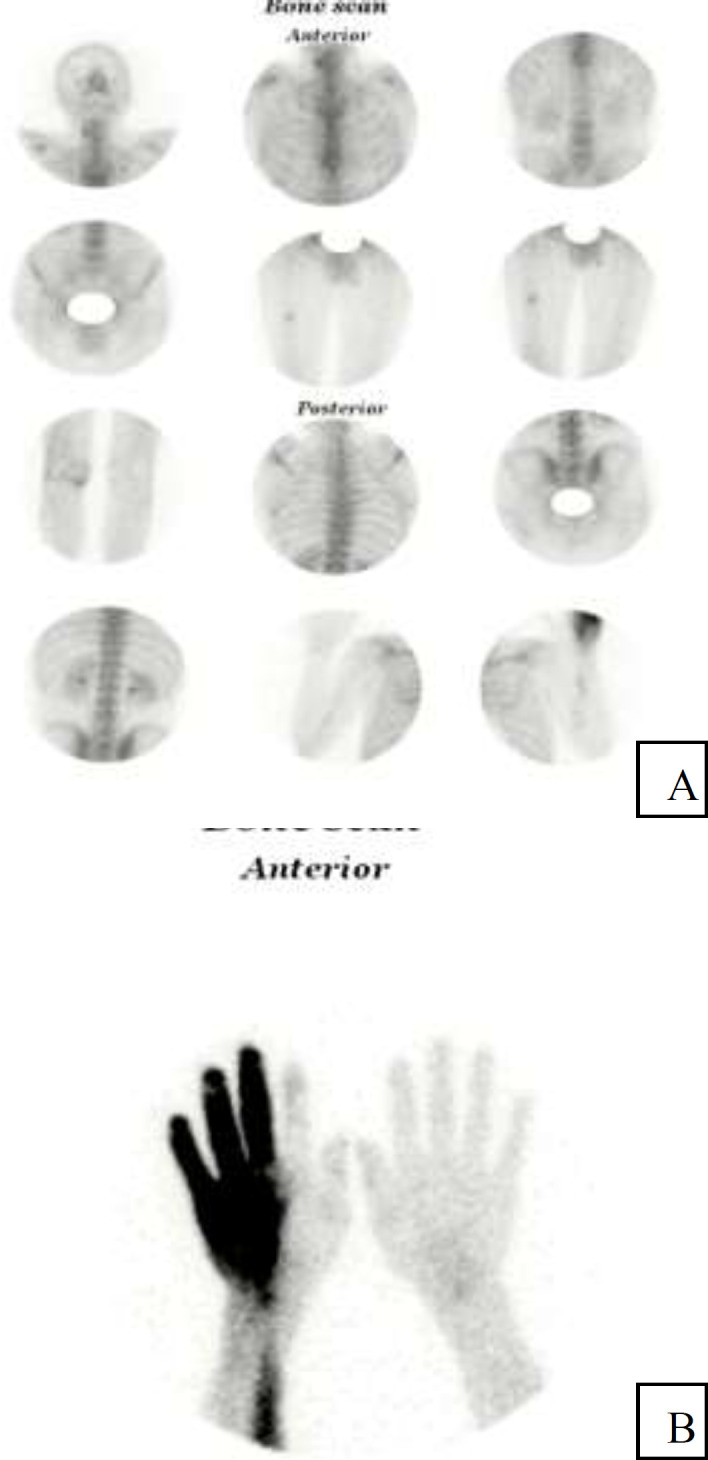
Whole body imaging (A) and static views (B) revealed diffusely increased uptake on the ulnar half of the right wrist-hand

A 44-year-old man with history of chest wall pain was referred to our nuclear medicine center for bone scintigraphy as part of a work-up for determining the pain source. The patient's pain had started 2 months ago and there was no history of trauma or fracture recently. The patient received an injection of 740MBq 99mTc-methylene diphosphonate (MDP) into a blood vessel at the right forearm. Two hours later, an increased uptake of activity was observed on the right forearm and ulnar half of the wrist-hand in the whole body and spot images (figure 1). Mild focal increased tracer uptake in mid shaft of the right femur was identified. There was no clinical history to explain this finding and further investigation with x-ray was requested.

## Discussion

Intra-arterial injection of a 99mTc labeled diphosphonate compounds such as Tc-99m MDP in the antecubital fossa into the brachial artery leads to in a glove-like pattern of dissemination of radiopharmaceuticals. After intra-arterial injection, radiopharmaceutical was circulated via the radial, ulnar, and interosseous arteries to the bones of the forearm, wrist, and hands and soft tissue distal to the antecubital fossa, creating the appearance of a glove (1). 

The effect of the first pass, when inadvertently injected into an artery, contributes to a different distribution pattern of the radiopharmaceutical. Radiopharmaceuticals are more likely to be uptake by the forearm or hand and cause the hot forearm or hot hand sign. The changes in image pattern are related to the involved artery and its perfusion (2). Because intra-arterial injection is accidental, unfamiliar images may cause misunderstanding and thus increase the suspicion of reflex sympathetic dystrophy (RSD).

 However, reflex sympathetic dystrophy, which mimics findings, should be excluded for differential diagnosis. Other differential diagnosis consists of frostbite damage and tourniquet effect (3, 4, 5). In our patient, there was no complaining of pain, paresthesia and numbness in the right wrist-hand and no clinical evidence of reflex sympathetic dystrophy was seen. Frostbite injury and tourniquet effect were also excluded by clinical history. 

Intra-arterial injection to the radial artery nearby the wrist may result to a different pattern of uptake because of arterial network anatomy of the hand. It was earlier termed as hot thumb and palm. In this case, bone scan shows significant increased uptake in the thumb and the rest of the metacarpals except distal half of the 5^th^ metacarpal (1). The hot hand-wrist appearance of our patient is different from the glove phenomena, which also results from injection of 99mTc-MDP into the ulnar artery in the antecubital fossa. 

The ulnar artery, a larger branch of the brachial artery, arises slightly below the elbow in the cubital fossa and inclines downward. It then reaches the wrist along the ulnar margin, crosses the transverse carpal ligament on the radial border of the pisiform bone, and instantly afterward is separated into two branches that arrive into the superficial and deep volar arches. The ulnar artery enters the palm and creates the superficial palmar arch, an important vascular organization in the hand. Common digital arteries are created from the superficial palmar arch to provide blood to the ulnar three digits. The injection into the right proximal ulnar artery produced our patient’s right-hand findings. The superficial palmar arch is formed predominantly by the ulnar artery, with a contribution from the superficial palmar branch of the radial artery. In other words, the superficial palmar arch is a continuation of the ulnar artery (the blood containing radiopharmaceutical) and supplies blood to the third, fourth, and fifth fingers via the digital arteries.

Thus, radiotracer from the right ulnar artery is deposited in the above-mentioned fingers as seen in our patient, explaining the hot right ulnar-sided three fingers. Besides the hot three digits, bone scan revealed increased uptake at the right forearm and hand which was significant on the ulnar half of the metacarpal region consistent with blood supplying from ulnar artery. No increased uptake was seen in the first and second metacarpals and fingers, because radial artery (the blood containing no radiopharmaceutical) is responsible for supplying blood to them. We report the interesting case of unintentional injection into the ulnar artery during bone scan and took a unique view of it. The scan findings were consistent with the anatomical and physiological expectations of the ulnar arterial perfusion range. This case shows that an injection with a bone-imaging radiopharmaceutical into the ulnar artery results in a hot ulnar half of the palm and ulnar-sided three digits, since these areas are directly exposed to the radiopharmaceutical, consequently, more radiopharmaceutical are deposited. Since, in our patient the specific view is seen as a hot half-hand, so this pattern is easily distinguished from the RSD, because the common pattern of the RSD is involvement of whole hand like a glove appearance. Physicians should be alert of intra-arterial injections before making a diagnostic assessment of asymmetric increased uptake, particularly if clinical information is insufficient. The injection site and vascular variations can even result in the different patterns of uptake (6). It is better to be cautious not to inject radiopharmaceutical into the arterial system and to avoid possible reassessment. We believe that good clinical evaluation is very essential before diagnosis. If a nuclear medicine technician is unsure about unintended intra-arterial injection, confirmatory three-phase imaging of the bone may be necessary for differentiation (7).

In conclusion a unique finding in this study is unintentional injection into the ulnar artery that has not been reported so far. It is important for the physician, and especially for the nuclear medicine technician to know the specific appearance created in the bone scan due to such an event so that they do not make a diagnostic mistake.
